# New Inventions

**Published:** 1895-12

**Authors:** 


### NEW INVENTIONS.

A SAFETY hitching-device, consisting of a rope, snap-hook, a series of perforated wooden blocks strung on rope, end to end, a spring between two blocks, the latter movable, so as to shorten or lengthen strap.

A detachable horseshoe, with upward flange around the toe, and two side-pieces with lugs; two ears at heels fastened by a bolt.

Veterinary mouth-speculum, arranged with jaws and locking device to maintain a fixed position of speculum. The means for locking are located at the extreme end of the jaws, so as not to cross the animal's mouth.

Horse ice-creepers, consisting of twin sections with spurs or calks, with means for adjusting to the shoe at the toe by flanges, and also at the heels.

A veterinary mouth-speculum, the upper and lower movable jaw pieces pivoted together at their near portions.

A paper-weight and calendar combined.

A horseshoe ice-calk with beveled inner edge, presenting a flaring upward seat and a spring calking, adapted to similar parts in the shoe.

A snap-hook, consisting of wire coiled at the base, and made to form a tongue as part of coil-spring.

A nailless horseshoe with side-clips and heel-lugs, a front lug with ratchet teeth, a bifurcated toe-pillar with serrated face toward hoof, with a strap from toe-pillar to under-heel projections.

A poultice, consisting of a mixture of linseed meal and sodium acetate, in a water-tight flexible cover.

A gaiting-device for horses, consisting of bars to be attached to shafts, a series of pulleys and hobbles for each limb, through which a rope is passed to move each of the feet.

